# Comparison of Objective and Subjective Indicators in Patients with Idiopathic Scoliosis Undergoing PSSE Therapy—Retrospective Observational

**DOI:** 10.3390/medicina62040652

**Published:** 2026-03-29

**Authors:** Marianna Białek, Sylwia Piorun, Ewelina Białek-Kucharska, Paulina Poświata, Małgorzata Poczynek, Justyna Pękala

**Affiliations:** 1 Functional Individual Therapy of Scoliosis (FITS Method), 59-411 Paszowice, Poland; vewelinav@o2.pl; 2 Aktis, 59-220 Legnica, Poland; spiorun@onet.pl; 3 FizjoUŚMIECH, 01-803 Warszawa, Poland; fizjousmiech@gmail.com; 4 FizMal, 59-300 Lubin, Poland; gosallegro@gmail.com; 5 Kinetik, 59-400 Jawor, Poland; biuro.kinetikjp@gmail.com

**Keywords:** idiopathic scoliosis, FITS Method, Physiotherapeutic Scoliosis-Specific Exercises (PSSE), Anterior and Posterior Trunk Symmetry Index (ATSI, POTSI), spinal deformity, Trunk Appearance Perception Scale (TAPS)

## Abstract

*Background and Objectives*: Physiotherapeutic Scoliosis-Specific Exercises (PSSE) are recognized treatment methods for idiopathic scoliosis, focused on correcting three-dimensional postural abnormalities. Objective indices such as Angle of Trunk Rotation (ATR), Anterior Trunk Symmetry Index (ATSI), and Posterior Trunk Symmetry Index (POTSI) enable precise assessment of clinical changes, while the Trunk Appearance Perception Scale (TAPS) reflects the patient’s subjective perception of their posture. Combining these data allows for a comprehensive assessment of the effects of therapy after intensive 5-day inpatient rehabilitation. We aimed to assess the improvement in the patients’ clinical appearance and compare objective and subjective trunk assessment indicators after intensive 5-day inpatient rehabilitation, treated by PSSE, according to the Functional Individual Therapy of Scoliosis (FITS) Method. *Materials and Methods*: This retrospective study included 75 patients with idiopathic scoliosis who participated in a 5-day inpatient rehabilitation, treated by FITS Method. The average age was 13.5 years, and 63% of the girls were after menarche. The mean Cobb angle was 27.41° in single-curve scoliosis and 31.03° in double-curve scoliosis (31.24° in the thoracic spine, 30.82° in the lumbar spine), Risser test 2, and ATR was 7.1° in the thoracic spine and 4.6° in the lumbar spine. Forty-nine patients wore a brace. At the beginning and end of inpatient care, objective assessments were performed, including ATR at the peak of the scoliosis using the Adams test and photoregistration of the trunk in the front and back standing positions—ATSI and POTSI. A subjective assessment was also performed using the TAPS. *Results*: A statistically significant difference was demonstrated after therapy in the ATSI (*p* < 0.001) and POTSI (*p* = 0.008) values. A reduction in the ATR in the thoracic spine was observed (*p* < 0.001). The TAPS questionnaire demonstrated a statistically significant difference in the values of all indicators measured before and after therapy: in the frontal plane SET 1 (*p* = 0.002), in the transverse plane SET 2 (*p* = 0.042), and in the frontal plane SET 3 (*p* = 0.028). A statistically significant negative correlation was demonstrated between objective and subjective indicators after therapy: ATR Th vs. TAPS-SET 2 (−0.45) (*p* < 0.001) and ATSI vs. SET 3 (−0.29) (*p* = 0.011). *Conclusions*: The subjective assessment of trunk appearance correlates with the objective assessment, except for SET 1 vs. POTSI. Patients who noticed a change in their posture can expect confirmation in objective clinical tests. FITS Method positively influences the improvement of subjective and objective assessments of idiopathic scoliosis patients during the short term of intensive care.

## 1. Introduction

Scoliosis is a three-dimensional spinal deformity that tends to progress during periods of rapid growth. The gold standard for idiopathic scoliosis management includes comprehensive diagnosis and treatment, in accordance with the current Society on Scoliosis Orthopaedic and Rehabilitation Treatment (SOSORT) recommendations [1]. Clinical and radiological examinations are key components of diagnosis. The Society recommends Physiotherapeutic Scoliosis-Specific Exercises (PSSE) and bracing. This treatment should meet the following criteria: prevention of scoliosis progression, patient-oriented approach, three-dimensional correction of the deformity, stabilization of the achieved correction, training in activities of daily living, and education of both patients and parents [2]. One of the methods meeting these criteria is Functional Individual Therapy of Scoliosis (FITS) [3,4,5,6].

Specific methods such as BSPT, SEAS, Schroth, Lyon, Side Shift, and FITS meet the SOSORT guidelines. Numerous publications confirm their effectiveness, demonstrating radiological and clinical improvements in studies with varying methodologies and follow-up times. Differences mainly concern the selection of equipment, therapeutic aids, and starting positions. The FITS Method takes a holistic approach to the patient (relaxing contracted muscles, examining and increasing shift, improving proprioception and core stabilization). It prepares the patient for correction, significantly simplifying, accelerating, and increasing the effectiveness of corrective exercises [3,4,5,6].

In patients with scoliosis, in addition to spinal deformity, three-dimensional postural alterations are observed, including asymmetry of the shoulders, scapulae, waist, and pelvis, as well as rib and/or lumbar prominence. These deformities may negatively affect patients’ self-esteem and quality of life. Therefore, conservative treatment should aim not only to correct the spinal curvature but also to address scoliosis-related clinical asymmetries of the trunk [1]. Effective therapy of idiopathic scoliosis is of considerable importance for the physical and psychological well-being of adolescents. In recent years, PSSE has been widely used in clinical practice as one of the principal conservative treatment methods, with reported positive outcomes [7].

Aesthetic concerns represent one of the major challenges faced by patients with scoliosis and can be assessed using both subjective and objective measures. Subjective evaluation is commonly performed using questionnaires such as the Trunk Appearance Perception Scale (TAPS) [8,9,10], Walter Reed Visual Assessment Scale (WRVAS) [11], Spinal Appearance Questionnaire (SAQ) [12], and Trunk Aesthetic Clinical Evaluation (TRACE) [13]. Objective assessment of posture is conducted using methods such as Anterior Trunk Symmetry Index (ATSI), Posterior Trunk Symmetry Index (POTSI) [14,15,16], Angle of Trunk Rotation (ATR) measurement using a Bunnell scoliometer and measurement of kyphosis and lordosis using an inclinometer. There are also other methods of objectively assessing the aesthetic profile of the examined person, including two-dimensional digital imaging and surface topography: nSpeck, ISIS, Quantec, and Formetric [17].

*Objectives*

1. To compare subjective and objective trunk assessment indicators in two planes (frontal and transversal) before and after therapy in patients treated by Functional Individual Therapy of Scoliosis (FITS).
2. To determine whether a 5-day intensive FITS program is associated with clinical improvement of a patient, confirmed by subjective and objective indicators.

## 2. Material and Methods

### 2.1. Study Design

A total of 75 individuals with idiopathic scoliosis were included in the retrospective observational study conducted in accordance with the Strengthening the Reporting of Observational studies in Epidemiology (STROBE) guidelines [18]. All subjects underwent a 5-day inpatient rehabilitation program based on the Functional Individual Therapy of Scoliosis (FITS Method). The data was collected from medical records from June 2022 to July 2024.

### 2.2. Participants

The inclusion criteria were consent to participate in the study, age 10–18 years, idiopathic scoliosis greater than 10 degrees, and participation in a FITS therapy session. Exclusion criteria included lack of consent, age under 10 years, three-curve scoliosis, functional, congenital, and neuromuscular scoliosis, and patients after surgical treatment. The study group comprised 61 girls (81.3%) and 14 boys (18.7%), with a mean age of 13.5 years ± 2.1. Menarche occurred in 63% of the females. The mean body mass was 48.07 kg, and the mean height was 162.47 cm. Single-curve scoliosis was identified in 17 patients, with a mean Cobb angle of 27.41° ± 10.19 in the range 10–50°. Double-curve scoliosis was present in 58 patients, with a mean Cobb angle of 31.03° ± 20.98 in the range 10–62°. In the thoracic region the mean Cobb angle was 31.24° ± 11.82, and in the lumbar spine it was 30.82° ± 10.98. The mean degree of skeletal maturity in the study group was 2.16 ± 1.97. Risser test indicated that 33 patients were classified at stage 0–1, 11 at stage 2–3, and 31 at stage 4–5. The mean Angle of Trunk Rotation (ATR) was 7.1° ± 4.1 in the thoracic region and 4.6° ± 3.7 in the lumbar region. A spinal brace was used by 49 patients (65.3%). The brace was worn according to medical recommendations and used for specific exercises during the treatment. The Anterior Trunk Symmetry Index (ATSI) was 26.2 ± 11, and the Posterior Trunk Symmetry Index (POTSI) was 25.8 ± 13.3 (Table 1).

The baseline Trunk Appearance Perception Scale (TAPS) questionnaire scores were 3.4 ± 0.8 in the frontal plane (SET 1—view from the back), 3.7 ± 0.7 in the transverse plane (SET 2—view in forward bend) and 3.4 ± 0.8 in the frontal plane (SET 3—view from the front).

### 2.3. Intervention

The children participated in a 5-day inpatient rehabilitation program consisting of specific physiotherapy exercises based on the FITS Method. The program aimed, among other aspects, to improve sensorimotor skills, increase trunk flexibility in the corrected scoliosis direction (shift and scoliosis derotation), enhance lower trunk stability and develop corrective patterns in functional postures across three planes. Additionally, the intervention included training in derotational breathing both with and without a brace, as well as three-dimensional autocorrection, defined as maintaining a corrected posture during activities of daily living [4,5,6]. Therapy was conducted for 3 h per day. Parents/caregivers actively participated in all sessions and were instructed in the correct performance of exercises to enable continuation of the therapy at home. Furthermore, both children and their parents attended an educational lecture on scoliosis and its treatment outcomes to increase their understanding of the condition and the therapeutic process.

**Figure 1:**
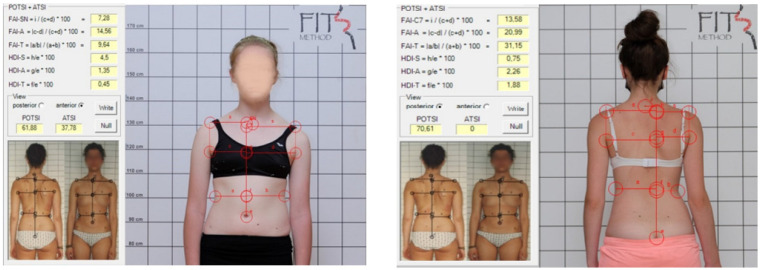
ATSI—frontal plane (view from the front); POTSI—frontal plane (view from the back).

At the beginning and end of the 5-day inpatient rehabilitation program, patients underwent a clinical assessment based on the FITS Method which included, among other measures, evaluation of the Angle of Trunk Rotation at the apex of the scoliotic curve using the Adams test [4,5,6], as well as photographic registration of the trunk in the standing position from anterior and posterior views. All measurements were conducted by the same two researchers: the first examiner consistently assessed the ATR, while the second examiner was responsible for all ATSI and POTSI measurements and data processing. The photographs were used to calculate trunk symmetry indices: ATSI and POTSI. After manually marking specific landmarks on the photographs in SCODIAC 2.6 software, the indices were automatically calculated. For ATSI, the following landmarks were identified: the jugular notch of the manubrium; the central point of the umbilicus; a bilateral point on the shoulder where the horizontal shoulder line intersects the vertical line extending from the axillary fold, the most prominent point of the anterior axillary fold; and the deepest waist indentation. For POTSI the following landmarks were identified: spinous process of C7; the top part of the gluteal cleft; bilaterally a point on the shoulder where the horizontal shoulder line intersects the vertical line extending from the axillary fold, the most prominent point of the posterior axillary fold; and the deepest waist indentation (Figure 1) [14,15,16].

**Figure 2:**
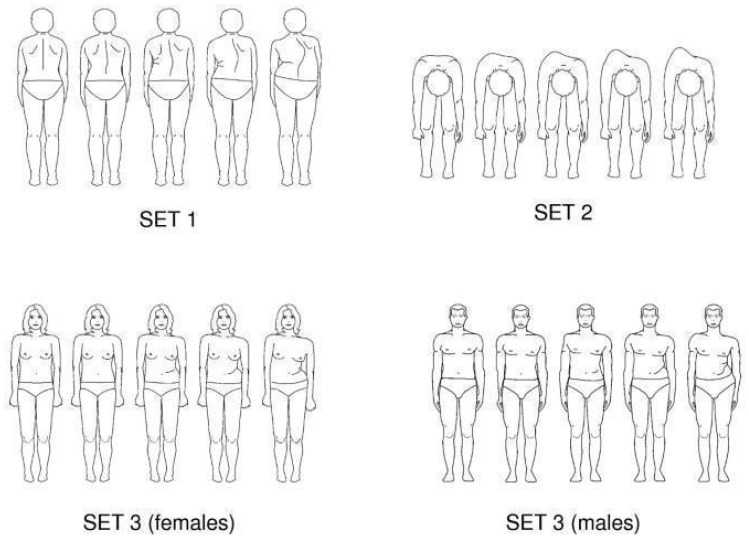
TAPS questionnaire.

Subjective evaluation was conducted using the TAPS [8,9,10]. This instrument is designed to assess the perception of trunk deformity by patients with idiopathic scoliosis. The scale enables patients to rate their perceived trunk asymmetry by comparing their own appearance with a series of drawings representing various degrees of asymmetry. Patients are instructed to select the drawing that best reflects their posture. TAPS comprises three sets of postural views: SET 1—posterior view in the frontal plane; SET 2—forward-bending view in the transverse plane; and SET 3—anterior view in the frontal plane, Figure 2.

**Figure 3:**
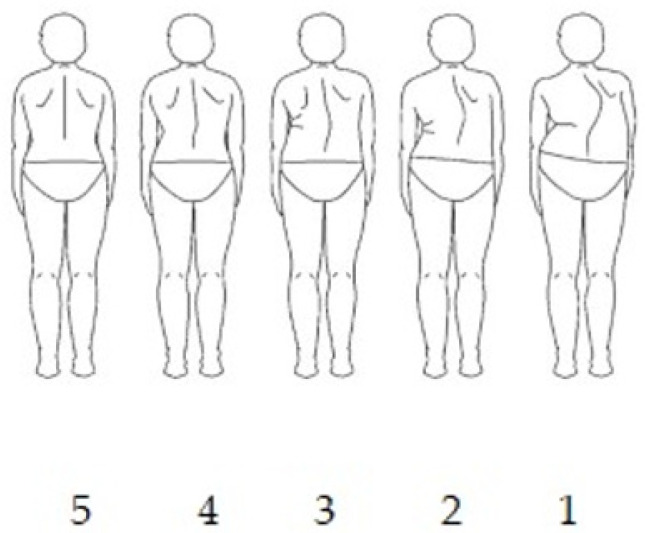
TAPS score range.

TAPS scores range from 1, indicating the most severe trunk deformity, to 5, indicating the least severe deformity. Higher scores reflect a better perception of trunk appearance, whereas lower scores indicate a poorer perception (Figure 3).

The children completed the TAPS questionnaire on the first and final days of the 5-day inpatient rehabilitation program. All necessary instructions for completing the questionnaire correctly were provided by a physiotherapist.

### 2.4. Statistical Methods

Statistical analysis of the collected material was performed using the Statistica 13.3 software package (StatSoft). Non-parametric tests were applied because the data did not meet the assumptions required for parametric testing, specifically normality of distribution, as verified using the Shapiro–Wilk test. For all variables, descriptive statistics were calculated, including the mean, median, minimum, maximum, and standard deviation. This study used means and standard deviations as they are reported to ensure transparency of the assessment. Correlations between two variables that did not meet the criterion of normal distribution were assessed using Spearman’s rank correlation coefficient. Variables with qualitative characteristics were analyzed using Pearson’s chi-squared test. The level of statistical significance was set at *p* < 0.05.

### 2.5. Ethical Considerations

The study was conducted in accordance with the Declaration of Helsinki and approved by the Ethics Committee of the Poznań University of Medical Sciences (number—212/24) on 6 March 2024 for studies involving humans. Patient confidentiality and data protection were ensured according to current international regulations [19]. Informed consent was obtained from participants and their parents/caregivers.

## 3. Results

### 3.1. Objective Evaluation Results

**Figure 4:**
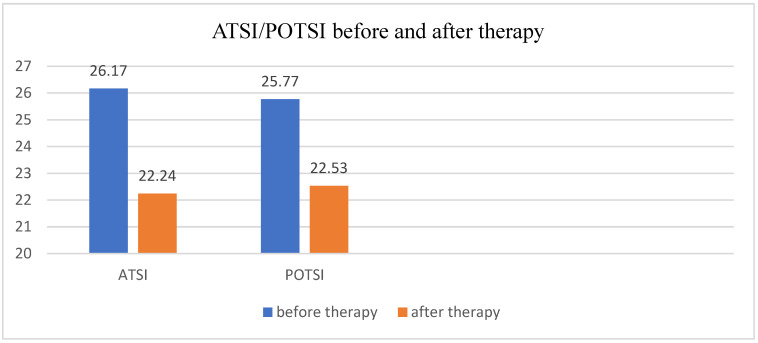
ATSI/POTSI values before and after therapy.

A statistically significant difference was observed in both Anterior Trunk Symmetry Index (ATSI) and Posterior Trunk Symmetry Index (POTSI) values when comparing measurements taken before and after therapy. ATSI improved significantly (*p* < 0.001) and POTSI also showed a significant improvement (*p* = 0.008) (Table 2, Figure 4). These findings indicate an improvement in trunk appearance following the 5-day inpatient rehabilitation, as lower ATSI and POTSI values reflect greater trunk symmetry.

Trunk symmetry analysis showed statistically significant post-therapeutic improvements in both ATSI (*p* < 0.001; ES = 0.36) and POTSI (*p* = 0.008; ES = 0.33) indices; with N = 75 these results confirm a consistent, small-to-moderate clinical effect of the intervention on postural alignment [20].

The therapy resulted in a statistically significant reduction in the Angle of Trunk Rotation (ATR) in the thoracic region (thoracic ATR; *p* < 0.001). An improvement was also observed in the lumbar region (lumbar ATR); however, this change was not statistically significant (*p* = 0.164) (Table 3).

Following the therapy, a statistically significant reduction in the thoracic ATR was observed (ES = 0.25; small effect size). By dedicating only a single examiner to do an ATR assessment, the standard error of measurement (SEM) was minimized and, according to the guidelines of Amendt et al., did not exceed 1 degree [20,21].

### 3.2. Results of the Subjective Study

Based on the Trunk Appearance Perception Scale questionnaire (TAPS), statistically significant differences were observed between before and after therapy values for all assessed indices: frontal plane—view from the back (SET 1; *p* = 0.002), transverse plane (SET 2; *p* = 0.042), and frontal plane—view from the front (SET 3; *p* = 0.028). The confidence interval (95% CI) for the subjective TAPS test was included—SET 1 (3.51–3.85), SET 2 (3.28–3.74) and SET 3 (3.39–3.92) (Table 4).

The analysis revealed statistically significant improvements across all three SET domains, with the most pronounced effect observed in SET 1 (*p* = 0.002; ES = 0.38). Both SET 2 (*p* = 0.042; ES = 0.26) and SET 3 (*p* = 0.028; ES = 0.25) also showed positive changes, confirming a consistent intervention impact on the studied characteristics with a small-to-moderate effect size [20].

### 3.3. Correlation of Objective and Subjective Indicators

A statistically significant negative correlation was observed between subjective and objective indices, with the exception of the POTSI vs. SET1 comparison. Higher subjective assessment scores (SET 1, 2, 3) were consistently associated with lower objective measurements (i.e., higher SET values corresponded to lower ATSI, POTSI, and ATR values). In other words, better clinical outcomes were accompanied by a more favourable patient perception of trunk appearance (Table 5).

No significant association was found between POTSI and TAPS—SET 1 scores, either before or after therapy (R = −0.01; *p* = 0.921 vs. R = −0.10; *p* = 0.409). These findings suggest that asymmetry of the posterior trunk surface has no significant impact on patients’ subjective assessment of trunk appearance.

The ATR in the thoracic region showed a statistically significant moderate negative correlation with TAPS—SET 2 scores, both before and after therapy (R = −0.45; *p* < 0.001 vs. R = −0.45; *p* < 0.001).

In addition, a weak but statistically significant negative correlation was observed between ATSI and TAPS—SET 3 scores both before and after therapy (R = −0.33; *p* = 0.004 vs. R = −0.29; *p* = 0.011), suggesting that asymmetry of the anterior trunk surface may influence patients’ perception of body appearance.

The results indicate that the subjective assessment of trunk appearance in TAPS—SET 2 shows the strongest correlation with ATR, whereas the indices of frontal asymmetry (ATSI and POTSI) demonstrate weaker or statistically insignificant correlations with TAPS—SET 3 and TAPS—SET 1. This finding suggests that patients’ perception of trunk appearance is biased towards transverse-plane deformity.

## 4. Discussion

Trunk deformity significantly affects posture assessment and appearance perception in patients with idiopathic scoliosis, mainly through the recognition of abnormal body proportions and trunk asymmetry [22]. Therapy should address both clinical symptoms and body perception to improve self-esteem and quality of life. A short-term pre–post design was used in this pilot study to assess the immediate response to the PSSE intervention. The lack of a control group is a limitation, but the short, 5-day follow-up period minimizes the influence of natural disease progression and standardized measurement protocols minimize bias. To reduce the risk of measurement errors and improper data interpretation, the therapeutic and diagnostic processes were conducted by a team of certified FITS Method therapists. To increase the reliability of the measurements, the same examiners performed the assessments of specific parameters at both the beginning and the end of the therapeutic camp. This approach allowed for the reduction of inter-observer variability.

In the present study, the authors compared subjective and objective indicators of trunk assessment in two planes: frontal-plane Trunk Appearance Perception Scale (TAPS)—SET 1 vs. Posterior Trunk Symmetry Index (POTSI), TAPS—SET 3 vs. Anterior Trunk Symmetry Index (ATSI), and transverse-plane TAPS—SET 2 vs. thoracic Angle of Trunk Rotation (ATR) before and after therapy. The obtained results can be compared with findings reported in the existing literature [23,24,25,26].

The study conducted by Topolovec evaluated 24 patients with idiopathic scoliosis treated using the Barcelona Scoliosis Physical Therapy School (BSPTS) Method for 60 min per day, five days per week, over a three-week period. The authors compared objective outcome measures ATSI, POTSI and ATR before and after therapy with subjective assessments of trunk appearance using the TAPS questionnaire. Following BSPTS therapy, a significant correlation was observed between ATSI and the total TAPS score (the mean score across all planes assessed in the TAPS test, i.e., SET 1 + SET 2 + SET 3). In contrast, no significant correlations were found between POTSI and the total TAPS score, or between thoracic ATR and SET 2. However, lumbar ATR demonstrated a statistically significant negative correlation with SET 2 after therapy [23].

The authors of the present study focused on direct correlations: TAPS—SET 1 (posterior view) with POTSI (posterior view), TAPS—SET 2 (forward bend) with ATR (forward bend), and TAPS—SET 3 (anterior view) with ATSI (anterior view). Consequently, only thoracic ATR and SET 2 scores are directly comparable. In our study, this relationship demonstrated a statistically significant negative correlation, in contrast to the findings reported by Topolovec, where no significant correlation was observed. Notably, lumbar ATR was not compared to SET 2 in this study, as the TAPS questionnaire depicts rib prominence associated with thoracic scoliosis only, and does not include lumbar prominence (lumbar scoliosis).

Belli et al. compared ATSI and POTSI with TAPS scores in the coronal plane. The recruitment phase lasted two months and the authors evaluated 15 patients with mild scoliosis, defined by a Cobb angle of up to 25 degrees, and found no statistically significant association between these indices (*p* = 0.2). The authors suggested that a low Cobb angle, combined with mild trunk deformity as assessed by ATSI and POTSI, may be insufficient to accurately predict patients’ perceived body image [24].

In the present study, involving patients with a mean Cobb angle of 27.41° (single-curve scoliosis) and 31.03° (double-curve scoliosis), a statistically significant correlation was identified between ATSI and SET 3 both before and after therapy. In contrast, no significant correlation was observed between POTSI and SET 1 at either time point. This result may be influenced by the short observation period and the fact that children usually look at their posture in the mirror from the front, rarely have the opportunity to see the back and therefore do not notice the existing deformations. Despite the absence of this correlation, patients reported a statistically significant improvement in their perceived posture following the 5-day inpatient rehabilitation program, as reflected by TAPS—SET 1 scores (*p* = 0.002).

In the transverse plane, Lendzion et al. reported a significant decline in self-evaluation of trunk aesthetics in patients with increased ATR. The study included 90 subjects based on the hospital records with a mean Cobb angle of 22.2° and a mean trunk rotation angle of 7.6, who completed the TAPS questionnaire and SRS-22. Patients were divided into two groups: one treated with bracing (35 subjects) and the other receiving physiotherapy alone (55 subjects). Analysis of the results demonstrated a negative correlation between total TAPS scores and both the Cobb angle and the angle of trunk rotation [25]. However, when SET 2 was analyzed specifically in relation to ATR, the correlation was weak and not statistically significant.

In the present study, a moderate statistically significant correlation was identified, indicating that greater ATR (reflecting increased rib prominence) was associated with poorer self-evaluation of appearance by the children.

Anna Podolska-Piechocka, during routine follow-up visits during brace treatment, evaluated 82 subjects with a mean Cobb angle of 31° and a mean ATR of 8.7, all of whom completed the TAPS questionnaire. The study demonstrated a moderate, statistically significant negative correlation between total TAPS scores and ATR, and between total TAPS and Cobb angle. Lower TAPS scores, indicating greater perceived deformity, were associated with higher ATR and Cobb angle values [26].

When analyzing the aforementioned studies, it should be noted that most authors consider only a single, averaged total TAPS score (SET 1 + SET 2 + SET 3) and relate it to a specific objective measure, such as ATR, ATSI, or POTSI. The authors of the present study believe that this approach may limit the precision of the analysis, as the posterior view (SET 1) reflects the coronal plane, whereas ATR represents deformity in the transverse plane, making a direct comparison difficult.

The authors of the present study also sought to determine whether a short period of intensive therapy using the FITS Method leads to clinical improvement, as reflected in both subjective and objective evaluation scores. They observed a statistically significant improvement in trunk appearance across all planes, as measured by the TAPS questionnaire. In contrast, the study by Topolovec reported no change in TAPS scores following therapy [23]. Misterska et al. evaluated trunk appearance in 36 female patients treated conservatively with Cheneau bracing, using the TAPS questionnaire at 6 and 12 months after treatment initiation. TAPS scores increased at each assessment, indicating a progressive improvement in patients’ perceived trunk appearance [27].

In addition to the improved TAPS scores, the authors of the present study also observed statistically significant improvements in ATSI and POTSI parameters. Comparable findings were reported by Chongow et al., who evaluated 128 patients with idiopathic scoliosis participating in a five-day training program according to the Schroth Method, conducted for 120 min per day in small two-person groups. They observed statistically significant improvements in both ATSI and POTSI [28]. Similar results were also reported by Topolovec.

The ATR also improved in both the thoracic and lumbar regions, which is consistent with the general principles of Physiotherapeutic Scoliosis-Specific Exercises (PSSE). In a 2025 meta-analysis, Jia et al. concluded that specific physiotherapeutic exercises are effective in reducing ATR [29].

The authors of the present study identified a negative correlation between objective and subjective indicators, specifically between SET 2 and ATR, and SET 3 and ATSI. They also demonstrated the effects of the FITS Method on the clinical appearance of patients with idiopathic scoliosis following a five-day inpatient rehabilitation program. Statistically significant improvements were observed in ATSI, POTSI, and ATR scores, alongside enhanced patient-reported trunk appearance as measured by the TAPS questionnaire.

### 4.1. Study Limitations

This study has several limitations: its retrospective design, potential variations in participants’ prior exercise or bracing history, short observation period, lack of sample size, and no control group.

### 4.2. Clinical Implications and Future Perspectives

This study indicates that short-term PSSE-FITS Method therapy affects the patient’s clinical appearance. It demonstrates improvements in postural symmetry in the frontal and sagittal planes (ATSI, SET 1), suggesting that this therapy can be used to restore trunk balance. Although the reduction in trunk rotation (ATR) is statistically significant, its smaller effect size indicates that structural changes in the spine require a longer treatment period. Analysis of the results showed that children’s subjective assessments do not always coincide with objective results. The ATSI, POTSI, and ATR tests allow us to monitor the therapeutic process, while subjective tests help determine the patient’s perception of their appearance. For a comprehensive therapeutic approach, it would be advisable to incorporate both methods of posture assessment. This study is retrospective and short-term in nature; therefore, it would be advisable to expand it to examine the long-term effects of the therapy.

## 5. Conclusions

1. Our findings demonstrate that a patient’s subjective self-assessment using the TAPS test does not necessarily correlate with objective clinical measurements. This suggests that a child’s perception of their trunk deformity is a highly individual matter and may not directly reflect the actual physical severity of the scoliosis.
2. Short-term intensive Functional Individual Therapy of Scoliosis (FITS Method) based on PSSE is associated with initial but statistically significant improvements in selected objective and subjective trunk appearance measures.

## Figures and Tables

**Table 1:** Descriptive statistics of the study group.

Parameters	Number (n = 75)	Average (SD)
Single-curve scoliosis (°)	17	27.41 ± 10.19
Double-curve scoliosis: (°)	58	31.03 ± 20.98
Th curve (°)		31.24 ± 11.82
L curve (°)		30.82 ± 10.98
Risser		2.16 ± 1.97
ATR Th (°)		7.1 ± 4.1
ATR L (°)		4.6 ± 3.7
ATSI		26.2 ± 11
POTSI		25.8 ± 13.3
Brace	49	

**Table 2:** ATSI and POTSI values before and after therapy.

	ATSI	POTSI
Before	26.17 ± 10.96	25.77 ± 13.28
After	22.24 ± 9.09	22.53 ± 10.81
Difference	3.93 ± 8.20	3.24 ± 9.51
	*p* < 0.001	*p* = 0.008

**Table 3:** Thoracic and lumbar ATR values before and after therapy.

	Thoracic ATR	Lumbar ATR
Before	7.12 ± 4.14	4.59 ± 3.66
After	6.08 ± 4.18	4.16 ± 3.27
Difference	1.04 ± 1.99	0.43 ± 2.31
	*p* < 0.001	*p* = 0.164

**Table 4:** SET 1, SET 2, and SET 3 values before and after therapy with confidence intervals (95% CI).

Average									
	SET 1	CI −95%	CI +95%	SET 2	CI −95%	CI +95%	SET 3	CI −95%	CI +95%
Before	3.36 ± 0.85	3.17	3.55	3.69 ± 0.73	3.53	3.85	3.43 ± 0.81	3.25	3.61
After	3.68 ± 0.77	3.51	3.85	3.88 ± 0.72	3.72	3.72	3.63 ± 0.73	3.47	3.79
Difference	0.32 ± 0.77	0.15	0.49	0.19 ± 0.67	0.04	0.34	0.20 ± 0.70	0.04	0.36
*p* before–after	*p* = 0.002			*p* = 0.042			*p* = 0.028		

**Table 5:** Statistical significance and correlations between the subjective TAPS (SET 1–3) assessment and the objective POTSI, ATSI, and ATR indicators.

	*p*	R
POTSI before–SET 1 before	0.921	−0.01
POTSI after–SET 1 after	0.409	−0.10
ATR Th before–SET 2 before	<0.001	−0.45
ATR Th after–SET 2 after	<0.001	−0.45
ATSI before–SET 3 before	0.004	−0.33
ATSI after–SET 3 after	0.011	−0.29

## Data Availability

The data presented in this study are available on request from the corresponding author due to legal and ethical reasons.

## References

[1] Negrini S., Donzelli S., Aulisa A.G., Czaprowski D., Schreiber S., De Mauroy J.C., Diers H., Grivas T.B., Knott P., Kotwicki T. (2018). 2016 SOSORT guidelines: Orthopaedic and rehabilitation treatment of idiopathic scoliosis during growth. Scoliosis Spinal Disord..

[2] Czaprowski D., Kotwicki T., Durmała J., Stoliński Ł. (2014). Physiotherapy in the treatment of idiopathic scoliosis—Current recommendations based on the recommendations of SOSORT 2011 (Society on Scoliosis Orthopaedic and Rehabilitation Treatment). Adv. Rehabil..

[3] Berdishevsky H., Lebel V.A., Bettany-Saltikov J., Rigo M., Lebel A., Hennes A., Romano M., Białek M., M’hango A., Betts T. (2016). Physiotherapy scoliosis—Specific exercises—A comprehensive review of seven major schools. Scoliosis Spinal Disord..

[4] Białek M., M’hango A. (2008). FITS concept—Functional Individual Therapy of Scoliosis. The Conservative Scoliosis Treatment.

[5] Białek M. (2011). Conservative treatment of idiopathic scoliosis according to the FITS Concept: Presentation of the method and preliminary, short term radiological and clinical results based on SOSORT and SRS criteria. Scoliosis.

[6] Białek M., M’hango A., Bettany–Saltikov J., Paz–Lourido B. (2012). Physical Therapy Perspectives in the 21st Century—Challenges and Possibilities.

[7] Liu L., Liu W., Xiang D., Wang J., Li X., Dong J., Xia H., Song X. (2025). An examination of factors affecting the efficacy of physiotherapeutic scoliosis-specific exercises in the management of adolescent idiopathic scoliosis. J. Intell. Med..

[8] Bago J., Sanchez-Raya J., Sanchez Perez-Grueso F.J., Climent J.M. (2010). The Trunk Appearance Perception Scale (TAPS): A new tool to evaluate subjective impression of trunk deformity in patients with idiopathic scoliosis. Scoliosis.

[9] Bago J., Matamalas A., Sánchez-Raya J., Pellise F., Pérez-Grueso F.J.S. (2018). Responsiveness of Image Perception Outcome Scales After Surgical Treatment of Idiopathic Scoliosis: A Comparison Between the Trunk Appearance Perception Scale (TAPS) and Scoliosis Research Society-22 (SRS-22) Questionnaire. Spine Deform..

[10] Matamalas A., D’Agata E., Sanchez-Raya J., Bago J. (2016). Trunk appearance perception scale for physicians (TAPS-Phy)—A valid and reliable tool to rate trunk deformity in idiopathic scoliosis. Scoliosis Spinal Disord..

[11] O’Sanders J., W’Polly D., Cats-Baril W., Jones J., Lenke J., O’Brien M.F., Stephens Richards B., Sucato D.J. (2003). Analysis of Patient and Parent Assessment of Deformity in Idiopathic Scoliosis Using the Walter Reed Visual Assessment Scale. Spine.

[12] Thielsch M.T., Wetterkamp M., Boertz P., Gosheger G., Schulte T.L. (2018). Reliability and validity of the Spinal Appearance Questionnaire (SAQ) and the Trunk Appearance Perception Scale (TAPS). J. Orthop. Surg. Res..

[13] Negrini S., Donzelli S., Di Felice F., Zaina F., Caronni A. (2020). Construct validity of the Trunk Aesthetic Clinical Evaluation (TRACE) in young people with idiopathic scoliosis. Ann. Phys. Rehabil. Med..

[14] Suzuki N., Inami K., Ono T., Kohno K., Asher M.A. (1999). Analysis of Posterior Trunk Symmetry Index (POTSI) in scoliosis. Stud. Health Technol. Inform..

[15] Stoliński Ł., Kozinoga M., Czaprowski D., Tyrakowski M., Cerny P., Suzuki N., Kotwicki T. (2017). Two–dimensional digital photography for child body posture evaluation: Standardized technique, reliable parameters and normative data for age 7–10. Scoliosis Spinal Disord..

[16] Stoliński Ł., Czeszejko-Sochacka E., Czaprowski D. (2022). The use of photoregistration in the objectification of body posture assessment (Wykorzystanie fotorejestracji w obiektywizacji oceny postawy ciała). Prakt. Fizjoterapia I Rehabil..

[17] Živković V.D., Dimitrijević L., Čolović H., Zlatanović D., Spalević M., Savić N. (2023). Aesthetic Appearance Assessment in Adolescents with Idiopathic Scoliosis. Acta Fac. Medicae Naissensis.

[18] von Elm E., Altman D.G., Egger M., Pocock S.J., Gøtzsche P.C., Vandenbroucke J.P. (2007). STROBE Initiative. The Strengthening the Reporting of Observational Studies in Epidemiology (STROBE) statement: Guidelines for reporting observational studies. Lancet.

[19] Official Journal of the European Union (2016). Regulation (EU) 2016/679 of the European Parliament and of the Council of 27 April 2016 on the Protection of Natural Persons with Regard to the Processing of Personal Data and on the Free Movement of Such Data, and Repealing Directive 95/46/EC (General Data Protection Regulation).

[20] Cohen J. (1988). Statistical Power Analysis for the Behavioral Sciences.

[21] Amendt L.E., Ause-Ellias K.L., Lundahl Eybers J., Wadsworth C.T., Nielsen D.H., Weinstein S.L. (1990). Validity and Reliability Testing of the Scoliometer. Phys. Ther..

[22] Savvides P., Gerdhem P., Grauers A., Danielsson A., Diarbakerli E. (2020). Self-Experienced Trunk Appearance in Individuals with and Without Idiopathic Scoliosis. Spine.

[23] Topolovec M. (2020). Effect of Scoliosis Specific Exercises on Trunk Asymmetry in Children with Adolescent Idiopathic Scoliosis (Učinak Vježbi Specifičnih za Skoliozu na Asimetriju Trupa Kod Djece s Adolescentnom Idopatskom Skoliozom). Ph.D. Thesis.

[24] Belli G., Toselli S., Latessa P.M., Mauro M. (2022). Evaluation of Self-Perceived Body Image in Adolescents with Mild Idiopathic Scoliosis. Eur. J. Investig. Health Psychol. Educ..

[25] Lendzion M., Łukaszewicz E., Waś J., Czaprowski D. (2018). Self-evaluation of Trunk Aesthetics in Conservatively Treated Children and Adolescents with Idiopathic Scoliosis. Ortop. Traumatol. Rehabil..

[26] Podolska-Piechocka A. (2013). Quality of Life of Patients with Idiopathic Scoliosis Treated with the Chêneau Brace (Jakość Życia Pacjentów ze Skoliozą Idiopatyczną Leczonych Gorsetem Chêneau—Rozprawa Doktorska). Ph.D. Thesis.

[27] Misterska E., Glowacki M., Latuszewska J., Adamczyk K. (2012). Perception of stress level, trunk appearance, body function and mental health in females with adolescent idiopathic scoliosis treated conservatively: A longitudinal analysis. Qual. Life Res..

[28] Chongov B., Alexiev V., Georgiev H., Kalinov K., Dimitrova E. (2017). Correlation between scoliosis deformity type and trunk symmetry before and after schroth physiotherapeutic exercises. Proc. Bulg. Acad. Sci..

[29] Jia Q., Zhang B., Wang H., Zheng W. (2025). Effectiveness of physical therapeutic scoliosis exercise (PSSE) intervention for adolescent idiopathic scoliosis: A systematic review and meta-analysis. BMC Musculoskelet. Disord..

